# BDNF Val66Met gene polymorphism modulates brain activity following rTMS-induced memory impairment

**DOI:** 10.1038/s41598-021-04175-x

**Published:** 2022-01-07

**Authors:** Kilian Abellaneda-Pérez, Pablo Martin-Trias, Catherine Cassé-Perrot, Lídia Vaqué-Alcázar, Laura Lanteaume, Elisabeth Solana, Claudio Babiloni, Roberta Lizio, Carme Junqué, Núria Bargalló, Paolo Maria Rossini, Joëlle Micallef, Romain Truillet, Estelle Charles, Elisabeth Jouve, Régis Bordet, Joan Santamaria, Simone Rossi, Alvaro Pascual-Leone, Olivier Blin, Jill Richardson, Jorge Jovicich, David Bartrés-Faz

**Affiliations:** 1 Medical Psychology Unit, Department of Medicine, Faculty of Medicine and Health Sciences, University of Barcelona, C/ Casanova, 143, 08036 Barcelona, Spain; 2 Institute of Biomedical Research August Pi i Sunyer (IDIBAPS), Barcelona, Spain; 3 CIC CPCET Service de Pharmacologie Clinique et Pharmacovigilance, CHU Timone, AP-HM, Marseille, France; 4 Department of Physiology and Pharmacology “V. Erspamer”, Sapienza University of Rome, Rome, Italy; 5 Department of Neuroscience and Neurorehabilitation, IRCCS S. Raffaele, Roma, Italy; 6 Istituto di Ricovero e Cura a Carattere Scientifico (IRCCS) SDN, Naples, Italy; 7 Neuroradiology Section, Radiology Department, Diagnostic Image Center, Hospital Clinic of Barcelona, University of Barcelona, Barcelona, Spain; 8 Magnetic Resonance Image Core Facility (IDIBAPS), Barcelona, Spain; 9 INSERM, Inst Neurosci Syst, Aix Marseille Université, 13005 Marseille, France; 10 Inserm, CHU Lille, U1171, Degenerative and Vascular Cognitive Disorders, University of Lille, Lille, France; 11 Sleep Unit, Neurology Department, Hospital Clinic of Barcelona, Barcelona, Spain; 12 Dipartimento di Scienze Mediche, Chirurgiche e Neuroscienze, Brain Investigation & Neuromodulation Laboratory (Si-BIN Lab), University of Siena, Siena, Italy; 13 Hinda and Arthur Marcus Institute for Aging Research and Deanna and Sidney Wolk Center for Memory Health, Hebrew SeniorLife, Boston, MA USA; 14 Department of Neurology, Harvard Medical School, Boston, MA USA; 15 Guttmann Brain Health Institute, Guttmann University Institute of Neurorehabilitation, Autonomous University of Barcelona, Badalona, Spain; 16 Neurosciences Therapeutic Area, GlaxoSmithKline R&D, Stevenage, UK; 17 Center for Mind/Brain Sciences (CIMEC), University of Trento, Trento, Italy

**Keywords:** Neuroscience, Cognitive neuroscience, Psychology, Human behaviour

## Abstract

The BDNF Val66Met gene polymorphism is a relevant factor explaining inter-individual differences to TMS responses in studies of the motor system. However, whether this variant also contributes to TMS-induced memory effects, as well as their underlying brain mechanisms, remains unexplored. In this investigation, we applied rTMS during encoding of a visual memory task either over the left frontal cortex (LFC; experimental condition) or the cranial vertex (control condition). Subsequently, individuals underwent a recognition memory phase during a functional MRI acquisition. We included 43 young volunteers and classified them as 19 Met allele carriers and 24 as Val/Val individuals. The results revealed that rTMS delivered over LFC compared to vertex stimulation resulted in reduced memory performance only amongst Val/Val allele carriers. This genetic group also exhibited greater fMRI brain activity during memory recognition, mainly over frontal regions, which was positively associated with cognitive performance. We concluded that BDNF Val66Met gene polymorphism, known to exert a significant effect on neuroplasticity, modulates the impact of rTMS both at the cognitive as well as at the associated brain networks expression levels. This data provides new insights on the brain mechanisms explaining cognitive inter-individual differences to TMS, and may inform future, more individually-tailored rTMS interventions.

## Introduction

Transcranial magnetic stimulation (TMS) is a non-invasive brain stimulation (NIBS) technique that can influence cognition in humans, including transient improvements^1^, as well as temporarily interferences^2^. In the context of NIBS research, neuroimaging information can be used both to guide stimulation^3^ and to reveal the TMS-induced effects on brain dynamics and their brain-behavior associations^4,5^. Regarding memory function, targeting the left dorsolateral prefrontal cortex (L-DLPFC) with brief and high frequency repetitive TMS (rTMS) trains during the encoding of visual stimuli resulted in subsequent memory recognition interferences^6–9^. Within the framework of a European Initiative (https://www.imi.europa.eu/projects-results/project-factsheets/pharma-cog), we previously provided first evidence of the replicability across centers of the effects of such a cognitive interference protocol using rTMS^10^. These results confirmed the potentiality of rTMS as a standardized easily replicable memory interference paradigm, within multicentric and large clinical trials. Nonetheless, significant intra and inter-individual variability in response to rTMS has been highlighted^4,10–14^. Genetic differences have been suggested as a critical contributing factor to NIBS-related variability^15^. In particular, the brain-derived neurotrophic factor (BDNF) Val66Met gene polymorphism, associated with individual differences in episodic memory^16,17^, hippocampal volumes^18,19^, and brain activity during motor learning tasks^20^, has also been related to the individual variability of TMS effects^21–26^ with some exceptions^27–29^. Specifically, when compared to Val/Val homozygous individuals, Met allele carriers showed a decreased susceptibility to TMS effects, with reduced or null facilitation and suppression effects of TMS investigations on motor cortical neurons^21,22,26^.

However, while the BDNF Val66Met gene polymorphism appears to influence cortical responses to TMS, the associated neural effects investigated so far have been mainly derived from neurophysiological measures within the motor system. In contrast, and despite the roles of this neurotrophin in synaptic plasticity and activity-dependent learning^30–32^, whether this polymorphism contributes to the individual differences observed in TMS-induced cognitive outcomes and its associated underlying brain mechanisms remains unexplored.

## Materials and methods

### Participants

The methods and experimental procedures (except for magnetic resonance imaging [MRI] procedures, see below) used in the present investigation are described in full in our previous report^10^. In brief, we adapted a TMS-memory interference protocol^6^ and demonstrated replicability of the induced effects across a two-center study. In the present report we focused on functional MRI (fMRI) activation changes during the recognition phase as a function of the BDNF Val66Met gene variation. From our previous study^10^, we included all participants with available fMRI data (N = 43; mean age: 23.47 ± 3.5 years). No additional subjects were recruited. All participants were healthy right-handed young males with at least secondary educational attainment. Subjects were recruited from two centers, Barcelona (BCN; N = 31) and Marseille (MRS; N = 12), where the experimental protocols had been harmonized in the context of the IMI FP7 European ‘Pharmacog’ research project (https://www.imi.europa.eu/projects-results/project-factsheets/pharma-cog). The study protocol was approved by the French ethics committee “SUD MÉDITERRANÉE I”, the French regulatory authority Agence Nationale de Sécurité du Médicament (ANSM) and the Spanish committee “Comitè d'Ètica d'Investigació Clínica de l’Hospital Clínic de Barcelona” (CEIC) in Barcelona. The study was in accordance with the Declaration of Helsinki. All volunteers were properly informed and gave written consent. Thereby, this study constitutes a sub-analysis of our previous investigation^10^, conducted within the IMI FP7 European ‘Pharmacog’ project, herein leveraging brain imaging and genetic data.

### Experimental design

**Figure 1:**
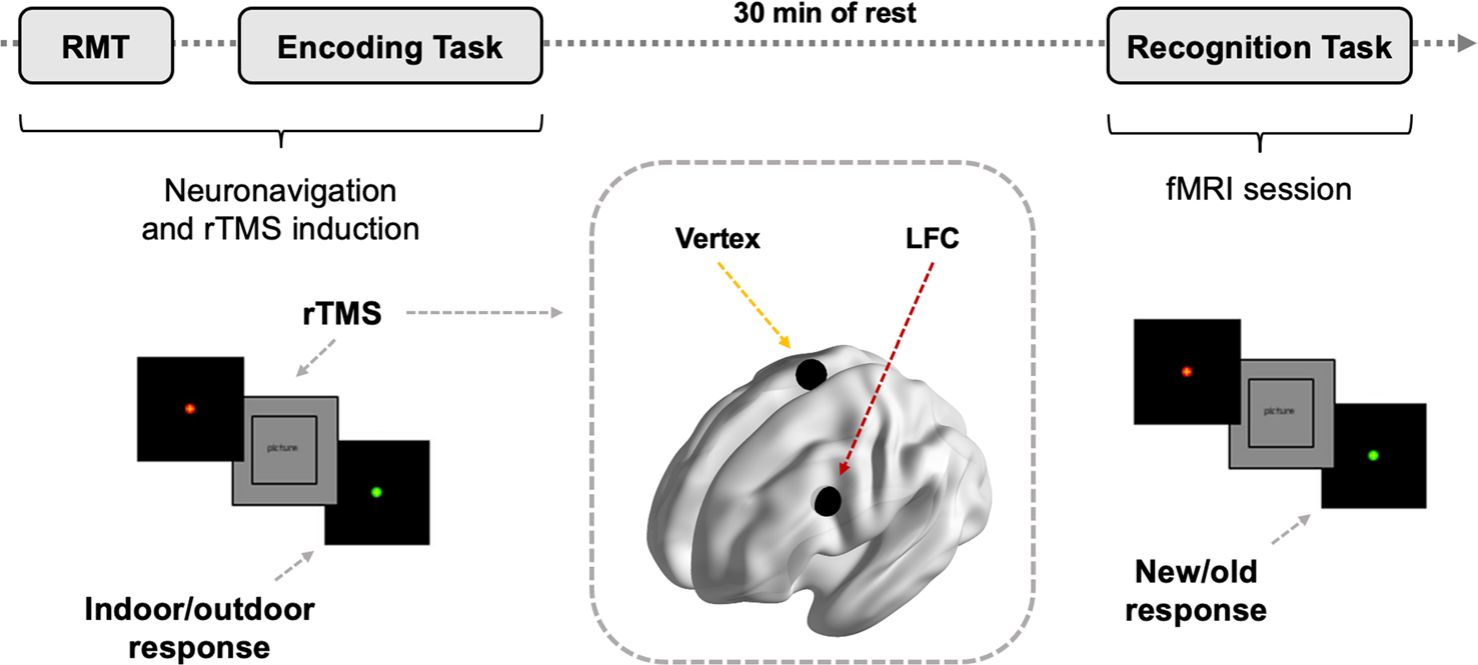
Study design overview. Participants underwent an encoding visual memory task while receiving rTMS over the LFC (MNI coordinates: X = − 42; Y = 10; Z = 30, according to Martin-Trias and colleagues^34^; pointed with a red arrow) or the vertex area (MNI coordinates: X = 4; Y = − 16; Z = 70, from Cz location according to Rojas et al.^68^, created for visual purposes, pointed with a yellow arrow). After a 30 min rest, participants completed the recognition of the visual memory task (i.e., discrimination between seen/not seen items in encoding phase) within the MRI scanner. RMT, resting motor threshold; rTMS, repetitive transcranial magnetic stimulation; LFC, left frontal cortex; fMRI, functional magnetic resonance imaging.

Participants attended their respective center (BCN or MRS) and undertook an encoding visual memory task while receiving short and high frequency rTMS trains (see next section for further detail) either over the left frontal cortex (LFC; experimental condition; see “TMS protocol” section) or the cranial vertex area (control condition). Subsequently, they performed the recognition of the visual memory task while brain imaging data was acquired (Fig. 1).

### TMS protocol

TMS was applied using figure-eight shaped coils. A MagPro X100 magnetic stimulator (MagVenture A|S, Denmark) combined with an eXimia Navigated Brain Stimulation system (Nexstim, Finland) and a Magstim stimulator (Magstim Company Limited, USA, CE certification) combined with the neuronavigation system Brainsight 2.2 (Rogue Research Inc., Montreal, QC, Canada) were used for the BCN and MRS subsamples, respectively. Resting motor threshold (RMT) was assessed as described in the International Standard Guidelines^33^ for all participants before conducting the encoding task. High frequency (20 Hz) 900 ms rTMS trains were delivered synchronously with the encoding task (500 ms after onset of pictures presentation) at 90% intensity of individual RMT. LFC stimulation site was extracted from a previous comparable fMRI memory study, as the maximum peak-activity coordinate within left frontal cortex (MNI coordinates: X = − 42; Y = 10; Z = 30^34^; see also Supplementary Material [SM] for anatomical details). Vertex stimulation control point was determined according to the 10–20 electrode placement (Cz^35^). Structural MRI was acquired for all participants (see below) and stimulation point was then transformed from MNI space to each native image using FLIRT tool from FSL (see below). To guide stimulation and ensure accuracy both in spot localization and coil positioning, neuronavigation was conducted in each volunteer on the basis of his structural MRI with stereotactic registration.

### MRI protocol

The MRI protocol had been harmonized amongst the different PharmaCog project centers^36^. Images were acquired using a Siemens Magnetom Trio Tim Syngo 3 Tesla system at the MRI Core Facility (IDIBAPS) of the Hospital Clinic of Barcelona, Barcelona, and with a Siemens Magnetom Verio 3 T at the Hôpital Timone in Marseille. All participants underwent fMRI acquisitions during the recognition memory task. Further, T1-weighted structural images were used for neuronavigation purposes (MRI parameters are detailed on SM).

### Memory task

The memory encoding task consisted of 6 blocks containing 12 pictures each (50% indoor, 50% outdoor). After a 30-min break, subjects performed the recognition memory task within the MRI scanner, in which they were shown 48 new pictures and 48 old pictures (24 previously encoded under LFC and 24 under vertex stimulation condition; see further details on SM).

### Genotyping

All participants underwent genotyping analysis of the functional polymorphism in the BDNF Val66Met gene, causing a valine (Val) to methionine (Met) amino acid substitution at codon 66. Results revealed that 19 participants were Met carriers (Met group; 18 heterozygous and 1 homozygous for the Met allele; Met allele frequency was 44.2% from the total sample), while 24 participants were homozygous for the Val allele (Val group; frequency of 55.8% from the total sample; see further information on SM).

### fMRI data

fMRI data from both centers was analyzed in a single site (BCN) with the FEAT-FSL (FMRIB’s Software Library v.6.00; http://fsl.fmrib.ox.ac.uk/fsl/^37^). After preprocessing of all individual fMRI scans, first-level analyses^38^ were individually customized, and three contrasts of interest: (1) mean hits frontal [HF]; (2) mean hits vertex [HV]; and (3) HF > HV were defined (see also SM). First level analyses were further fit into higher-level statistics using the FMRIB's Local Analysis of Mixed Effects (FLAME^39^). For the main contrast of interest (i.e., HF > HV), we created group GLM designs to evaluate: (1) differences between BDNF subgroups (Val vs. Met); and (2) subgroups mean activity maps. Additionally, group differences (Val vs. Met) were tested for the other first-level contrasts calculated (i.e., mean HF and mean HV; see SM for further details). All analyses were performed in the whole brain at a voxel-wise level, and a Z > 2.3 was used to define contiguous clusters of activity, then cluster significance levels were estimated and corrected using family-wise error (FWE) correction. The significance threshold was set at a corrected p < 0.05.

### Statistical analysis

Non-imaging data analyses were performed using IBM SPSS (IBM Corp. Released 2020. IBM SPSS Statistics for Windows, Version 27.0. Armonk, NY: IBM Corp). For cognitive performance data, accuracy (percentage of pictures correctly categorized as indoor or outdoor), and reaction time (RT; time elapsed from the presentation of a picture to the subsequent response) during encoding and hits (percentage of correctly recognized pictures), and RT (time elapsed between picture appearance and yes/no motor response) during recognition, were collected for both experimental conditions (LFC and vertex). For these outputs, one-way repeated measures ANOVAs were conducted with experimental conditions (i.e., LFC vs. vertex) as within-subject factor and BDNF Val66Met gene polymorphism (i.e., Val vs. Met) as between-subject factor. When interactions or main effects emerged, subsequent pair-wise analyses were conducted to investigate directionality of the data, or to explore if a specific group or condition driven the observed results. Hence, paired-samples t-test analyses were performed comparing the performance between experimental conditions in each genetic subgroup. Additional independent-samples t-test analyses were conducted comparing genetic subgroups in each experimental condition performance’ measure. Finally, we extracted the mean values of the blood oxygen level dependent (BOLD) within the significant regions of interest (ROI) derived from the fMRI analyses, in order to test associations between functional activity and cognitive performance estimates using Pearson correlations. All statistical analyses were two-tailed and α was set at 0.05 (see SM for further details and analyses). Due to technical issues during the recording of the encoding phase, data from three subjects was not available for conducting accuracy statistical analyses (see also Table S1).

## Results

### Cognitive performance

**Figure 2:**
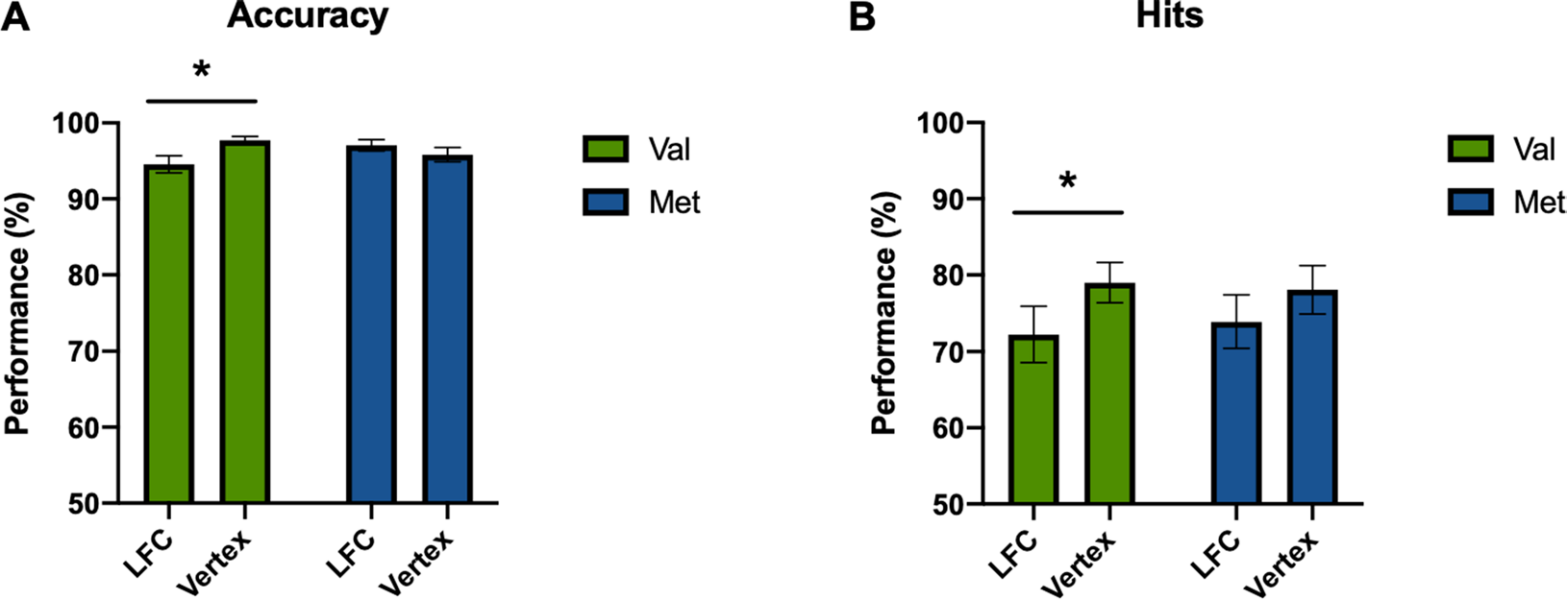
TMS effects on cognition as a function of BDNF Val66Met gene polymorphism. Cognitive performance results considering (**A**) accuracy during encoding as well as (**B**) hits during recognition. Statistical analyses were performed via one-way repeated measures ANOVAs with experimental conditions as within-subject factor and BDNF Val66Met gene polymorphism as between-subject factor. Subsequent pair-wise analyses were conducted with t-tests. * Significant differences (p < 0.05). LFC, left frontal cortex.

During encoding, an interaction between condition (LFC vs. vertex) and BDNF Val66Met gene polymorphism (Val vs. Met) factors was found for accuracy (*F*_*(1,38)*_ = 7.397, *p* = 0.010, *η*_*p*_^2^ = 0.163). Statistically significant main effect did not emerged (*F*_*(1,38)*_ = 1.417, *p* = 0.241, *η*_*p*_^2^ = 0.036). Subsequent paired-samples t-test analyses revealed that accuracy was higher following vertex as compared to LFC stimulation for the Val/Val allele carrier subgroup (*t*_*(21)*_ =  − 2.686, *p* = 0.014), but not for the Met allele carriers (*t*_*(17)*_ = 1.166, *p* = 0.260; Fig. 2A).

For the recognition memory, a main effect of condition (LFC vs. vertex) was found for hits (*F*_*(1,41)*_ = 8.924, *p* = 0.005, *η*_*p*_^*2*^ = 0.179), but not an interaction (*F*_*(1,41)*_ = 0.507, *p* = 0.481, *η*_*p*_^*2*^ = 0.012). Subsequent paired-samples t-test analyses revealed that hits performance was significantly higher following vertex as compared to LFC stimulation for the Val/Val allele carrier subgroup (*t*_*(23)*_ = − 2.865, *p* = 0.009), but not for Met allele carriers (*t*_*(18)*_ = − 1.471, *p* = 0.159; Fig. 2B). Following independent t-test analyses showed no differences in each experimental condition when comparing the BDNF polymorphisms both regarding accuracy and hits (accuracy, LFC: *t*_*(38)*_ = − 1.744, *p* = 0.089; vertex: *t*_*(38)*_ = 1.873, *p* = 0.069. hits, LFC: *t*_*(41)*_ = − 0.327, *p* = 0.756; vertex: *t*_*(41)*_ = 0.226, *p* = 0.822).

Regarding RT, a main effect of condition was observed for accuracy’ RT (*F*_*(1,38)*_ = 5.240, *p* = 0.028, *η*_*p*_^*2*^ = 0.121), but not an interaction (*F*_*(1,38)*_ = 3.658, *p* = 0.063, *η*_*p*_^*2*^ = 0.088). Subsequent paired-samples t-test analyses revealed that the accuracy’ RT was lower following vertex as compared to LFC stimulation for the Met allele carrier subgroup (*t*_*(17)*_ = 2.365, *p* = 0.030), but not for Val/Val allele carriers (*t*_*(21)*_ = 0.348, *p* = 0.731). These differences did not survive when controlling RT for accuracy performance (interaction: *F*_*(1,38)*_ = 0.277, *p* = 0.602, *η*_*p*_^*2*^ = 0.007; main effect: *F*_*(1,38)*_ = 0.003, *p* = 0.958, *η*_*p*_^*2*^ < 0.001; paired samples t-test analyses: Met carriers: *t*_*(17)*_ = 0.308, *p* = 0.761; Val/Val carriers: *t*_*(21)*_ = − 0.492, *p* = 0.628). Regarding hits’ RT, neither significant interaction (*F *_*(1,41)*_ = 0.145, *p* = 0.706, *η*_*p*_^*2*^ = 0.004) nor main effect (*F*_*(1,41)*_ = 0.584, *p* = 0.449, *η*_*p*_^*2*^ = 0.014) was found (please, refer to SM for sanity checks and to see Table S1, reporting all behavioral data, and Fig. S1, showing individual data).

In summary, at the cognitive level we observed greater rTMS interference effects amongst Val/Val individuals compared to Met allele carriers, both at the encoding as well at the recognition phase.

### fMRI findings

**Figure 3:**
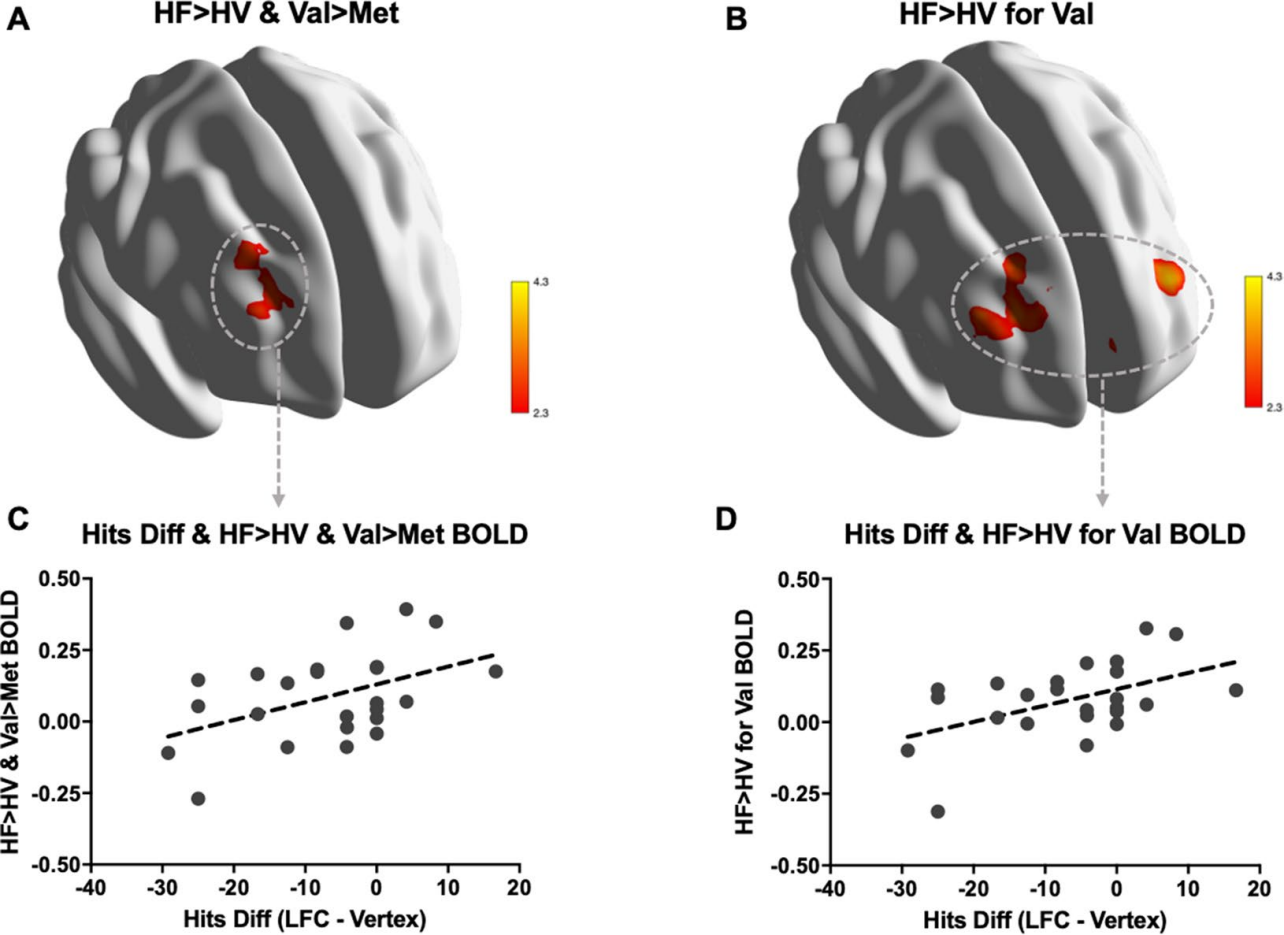
TMS effects on brain activity as a function BDNF Val66Met gene polymorphism and its relationships with cognitive performance. (**A**,**B**) fMRI activity maps for the HF > HV contrast at both group differences (Val > Met) and mean Val group. (**C**,**D**) Scatter plots showing Pearson correlations between hits difference (i.e., LFC hits—vertex hits) and BOLD signal values within the ROIs displayed in (**A**,**B**), only considering the Val group. HF, hits frontal cortex; HV, hits cranial vertex; Diff, difference; LFC, left frontal cortex; BOLD, blood oxygen level dependent.

First, we investigated whether there were fMRI BOLD activation differences for memory performance (hits) associated to both rTMS conditions (HF and HV) as a function of genetic background (Val vs. Met). For the contrast of interest (i.e., HF > HV), we found BDNF group differences in the sense of more fMRI BOLD activation for the Val/Val than the Met allele carrier subgroup over a right frontal cluster comprising the frontal pole and the middle and superior frontal gyri (Fig. 3A). Subsequent analyses corroborated that these effects were driven by increased frontal fMRI BOLD activation amongst Val/Val subgroup during memory recognition for items corresponding to the LFC condition relative to vertex. The clusters identified were allocated bilaterally including the frontal pole, the paracingulate gyrus, and the anterior division of the cingulate gyrus (Fig. 3B). No significant differences appeared for the Met subsample in the same HF > HV contrast of interest. All these results survived when controlling for the center variable (no effect of center was observed). Additional analyses exploring genetic group fMRI differences during both mean HF and mean HV contrasts can be found in SM and Fig. S2.

### Brain-behavior associations

Finally, in order to assess the behavioral significance of the fMRI results, additional analyses were performed using individual BOLD signal values in the HF > HV contrast in both genetic groups. For the mean activity values within the significant result derived from the HF > HV & Val > Met imaging contrast (Fig. 3A, in red), a positive correlation with Hits’ difference (i.e., HF-HV) in the Val group was observed (*r* = 0.462, *p* = 0.023; Fig. 3C). Further, in the HF > HV significant regions for Val/Val subgroup (Fig. 3B, in red), another positive correlation with Hits’ difference was detected (*r* = 0.499, *p* = 0.013; Fig. 3D). The associations between the behavioral measure and the means of activity within the stated imaging clusters did not reach statistical significance in the Met group (*r* = 0.445, *p* = 0.056; *r* = 0.453, *p* = 0.052). Further exploratory correlative imaging-behavior analyses can be found in SM. Hence, those subjects in the Val group (which, at the cognitive level, were more vulnerable to rTMS interference than the Met allele carrier subgroup) exhibiting greater over-activations in the stated frontal brain areas, were those in whom the TMS-related cognitive interference was lower.

## Discussion

The present study confirmed the expected episodic encoding interference using an adaptation of a rTMS memory paradigm^6–9^. Specifically, we found that cognitive interference effects were driven by the Val/Val homozygous. Furthermore, this effect was observed in the context of a greater brain activity pattern during the LFC condition in Val/Val subjects, which in turn was positively associated with memory performance. Present results, therefore, are consistent with the BDNF Val66Met genetic variation being a relevant factor for inter-individual variability of rTMS effects on cognitive function, and provides novel evidence of the underlying neurophysiological mechanisms subtending such effects.

### Cognitive findings

Cellular and molecular studies have stablished that BDNF exerts an important role in regulating the functional dynamics of brain regions relevant for learning and memory^40^ and that the Met allele of the BDNF Val66Met genetic variation is associated with a decreased activity-dependent secretion of BDNF^18^. Early influential findings (e.g.,^18^), indicated that Met over Val/Val allele carriers exhibited lower memory performance. However, a recent systematic review observed that only around half of studies reported positive associations, leading the authors to conclude that the effects of this genetic variation on human cognition remain inconclusive^40^.

Previous inconsistent findings may obey to several conditions, including sample characteristics (i.e., sample sizes and healthy individuals vs. patients^40^, age effects^41^, or specific cognitive tasks^42^). In our study, we did not find a difference in memory recognition dependent on Val/Met variation when groups were compared under the control condition. However, we did observe a clear genetic effect when memory was assessed as a function of rTMS interference. Therefore, present results highlight that using rTMS as a perturbation approach^43^ represents a sensitive approximation to reveal the effects of factors contributing to individual differences in cognitive performance that may not be apparent during conventional cognitive assessments.

More specifically, our findings emphasize that rTMS delivered over the LFC decreased performance in Val/Val carriers both during the initial attentional tasks as well as at subsequent memory recognition performance. While memory effects have been consistently reported in the literature using the original interference paradigm (i.e.,^6^) the effects during the encoding process were not reported previously. A possibility regarding this discrepancy may be related to the specific stimulation site used in our adaptation of the protocol. Here, and guided by a previous fMRI pilot study^34^, our stimulation target overlapped with the left frontal eye field (L-FEF) node of the dorsal attention network (DAN), which is associated to the capacity of orienting one’s focus to a particular task and specifically contributes to top-down guided voluntary allocation of attention to locations or features^44–46^. In contrast, F3 site of the 10–20 EEG system, used in the original studies^6–9^, is mainly located within the frontal areas of the left executive-control network (LECN), which subtends executive functions including working memory and decision-making processes (e.g.,^47^; for a visual comparison of stimulation sites, see Fig. S3). Hence, the effect we observed at encoding accuracy could be explained by a more specific rTMS-induced perturbation over dorsal attention systems. In these regards, it can be conceived that the TMS-induced modulation at the attentional memory stage constrained the behavioral TMS effects observed during the retrieval, and therefore in the current study are difficult to dissociate from its preceding codification phase.

Altogether, our findings align with previous literature conducted with electromyographic recordings of MEPs during stimulation of the motor cortex (e.g.,^22,48^) as well as with a previous report focusing on decision-making processes^49^, confirming that BDNF Val66Met genetic polymorphism is a relevant factor explaining variability in response to cognitive effects of TMS, and suggesting greater susceptibility for Val/Val homozygous.

### Brain activation findings

Previous neuroimaging studies conducted during memory tasks revealed greater medial temporal lobe (MTL) activity amongst Val/Val allele carriers^17,18^ or similarly, negative associations between brain activity and the dose of Met-BDNF allele^50^. In the same line, during a working memory task, greater brain activity with increasing memory loads in the medial prefrontal areas, in particular within the superior frontal gyrus, was observed amongst Val/Val but not Met carriers^51^. However, the directionality of these findings is not consistent across the studies, as for example Dennis et al.^52^ reported greater MTL activity amongst Met/− allele bearers.

As in the cognitive studies referred above, a number of factors, including the type of memory task employed (i.e., item vs. relational), design (i.e., blocks vs. event-related) or differences in performance between genetic groups may determine the significancy and directionality of the observed functional imaging findings. Furthermore, it should be noted that a meta-analysis on this topic concluded that while a biological effect of this genetic variation detectable in humans is plausible, its analysis using imaging protocols will require large samples sizes to identify consistent effects^53^. In our study, with a comparable or larger sample size than previous investigations, we did not observe differences between genetic subgroups in the control condition. However, differences were clearly amplified when reflecting recognition memory performance for items where rTMS interference was introduced at encoding. Hence, aligned with our conclusions at the cognitive level, present findings reinforce the idea that rTMS combined with neuroimaging techniques represents a useful experimental approach to highlight the underlying brain mechanisms through which factors that influence variability of cognitive functions in humans operate.

Across the different imaging analyses, present findings revealed that Val/Val subjects exhibited a more extended pattern of brain activity than Met group after rTMS over the LFC. First, increased activity in parietal, temporal, occipital and motor regions were found corresponding to LFC-linked recognition events (Fig. S2A). Topographically, some of these areas are embedded within the general brain network subtending memory recognition processes^54^ or have been observed during visual working memory fMRI studies (e.g.,^55^). The pattern also included areas typically non-related to episodic memory processes, such as pre and posterior central gyri regions contralateral to the hand used to press the ‘correct’ (i.e., hits) button during the task. When looking at more specific effects, we identified increased brain activity over the right frontal region for the HF > HV contrast. In subsequent analyses conducted to explore the directionality of such results, we observed that Val/Val group (but not Met carriers) showed greater activity specific to rTMS-LFC (i.e., HF > HV) in bilateral frontal and anterior cingulate regions. These findings indicate that increased bilateral frontal activity, and particularly in contralateral areas to the stimulation site (i.e., rTMS was delivered at encoding over the LFC), was observed amongst Val/Val subjects and that this was only evident during the effortful memory recognition process where, in average, rTMS induced greater cognitive deficits in Val compared to Met group. In this sense, increased brain activity in frontal regions including bilateral activations (i.e., more widespread allocation of functional resources) have been frequently reported in the neuroimaging literature, in particular attributed to compensatory processes (see below). Furthermore, previous rTMS findings revealed that amongst young but not older individuals^7^, perturbation of brain activity over the right frontal cortex during memory recognition results in clear interference effects, advocating for a causal role of this region during these cognitive processes^6–9^. Overall, present fMRI findings suggest that the greater permeability of Val/Val subjects to rTMS effects during memory encoding resulted in over recruitment of brain regions specifically subtending memory recognition.

### Associations between fMRI findings and cognitive performance

We found that the observed increases of brain activity amongst Val/Val allele carriers were positively associated with memory performance. This was evidenced in a specific way through right and bilateral frontal activations, after subtracting both brain activity and memory performance related to the vertex control condition from LFC (Fig. 3C,D, respectively). Also, in a general manner, brain activity under the LFC condition was associated to hits performance (Fig. S2B). Such findings suggest evidence of compensation, a concept particularly developed within the field of cognitive neuroscience of aging, and which refers to the capacity of engaging additional neural resources (i.e., such as reflected by increased fMRI BOLD signal) to counteract the lack of functionality of the typical brain resources in a given situation^56–58^. Compensation has been frequently associated with increases in contralateral recruitment as an adaptative shift^57–59^ and is a term associated to enhanced cognitive performance. However, a task-related increase in cognitive demands may be completely counteracted by the recruitment of additional neural resources, or it may reduce but do not completely eliminate the gap between task demands and available resources^57^. This latter scenario, which may also be conceptualized under the term ‘attempted compensation’^60^, appears to fit particularly well with our findings of positive associations between the expression of neural resources amongst Val/Val allele carriers, within the context of an overall greater rTMS-induced interference on memory recognition, as compared to Met/− individuals.

Compensatory processes have also been reported in the context of task-performance studies combining TMS with functional neuroimaging data. For example, in the language domain, bilateral reorganizations in homologous right-hemispheric areas after disruption of left-hemispheric language regions have been reported (reviewed in^61^). In the case of memory studies, rTMS-induced bilateral fronto-parietal reorganizations amongst older individuals concomitant with an improvement in a visual memory task were reported^62^. Furthermore, Davis and colleagues^59^ delivered 1 and 5 Hz rTMS trains over the left middle frontal gyrus and investigated the effects during a subsequent fMRI source memory task. First, they observed that 1 Hz, an inhibitory stimulation, resulted in decreased ‘memory-related’ activity in the prefrontal area under stimulation while 5 Hz, an excitatory protocol, increased it. Second, and using multivariate network analyses, the authors found that induced decreases in the stimulated node (i.e., following 1 Hz rTMS) resulted in memory-related increases in connectivity with other cortical regions (frontal, temporal, parietal), in particular with homologous contralateral right PFC areas, thus providing direct support for the compensatory hypothesis (i.e., that local dysfunction is counterbalanced by a more global recruitment; for a review on this topic, see also^63^).

The present findings using rTMS as a technique to directly manipulate neural activity are aligned with previous rTMS studies conducted amongst older adults (i.e.,^59,62,64^) showing that a disruption of local resources resulted in a shift of brain activity positively associated with task performance. However, it should be noted that in the present study we employed an rTMS ‘online’ stimulation protocol during the memory encoding phase, using very brief (i.e., 900 ms) trains of a high frequency stimulation (20 Hz), that in principle should not induce after-effects. Hence, and since our memory recognition task was conducted 30 min later, it is very unlikely that the observed fMRI findings are confounded by direct TMS effects (i.e., ‘after-effects’) on the reorganization of brain networks. Therefore, our fMRI findings reveal genuine differences on brain network expression directly associated to the BDNF Val66Met human variation. Nonetheless, it cannot be ruled out the possibility that our results, focused on functional systems, might be partially explained by brain structural differences related to the BDNF Val66Met polymorphism. In this vein, previous studies have claimed that a critical role of the BDNF polymorphism on brain plasticity might be associated with the robustness of white matter structural networks, being Val homozygote subjects those showing more resilience under targeted attacks on central nodes^65^. However, in a more general manner, it should be noted that structural and functional connectivity relationships appear to be weak in healthy populations^66^. Altogether, and according to strong cellular and molecular evidence linking this polymorphism with synaptic transmission and brain plasticity mechanisms^32^, the most likely explanation for the observed overactivations suggest the expression of rapid adaptative plasticity responses amongst Val/Val allele carriers allowing to−partially−counteract greater interference effects at encoding.

## Limitations

Our study has some limitations. First, in the present investigation, all subjects were male. This fact, which limits the generalization of our results, is clearly acknowledged and was chosen because an eventual objective of our research, within the framework of the European IMI-PharmaCog European initiative, was to compare our TMS findings with those obtained using sleep deprivation, as a second planned controlled sets of experiments designed to transiently disrupt memory processes. Gender effects have been described to interact with sleep restriction in their impact on cognitive performance^67^, and therefore only male participants were included in all studies. Further, and due to its low relative occurrence, we could not analyze event-related brain activity differences associated to the induced errors in memory performance (i.e., misses or false alarms). Finally, it should be noted that albeit a direct perturbation role of rTMS can be assumed for the interference of memory encoding process, the proof that overexpression areas are reflecting compensatory efforts (i.e., causally associated with task performance) would equally require to target such areas/networks with an additional right-sided TMS perturbation approach and observe the behavioral effects.

## Conclusion

Through the induction of encoding interference, the present rTMS-fMRI investigation allowed to increase evidences of the study of genetic influences on the expression of brain networks involved in memory functions. Present results also suggested that Val/Val individuals may be particularly sensitive to TMS effects, indicating that future studies with individuals bearing this genetic profile could be a useful approach to detect proof of concept evidence of TMS effects on memory in humans, including those investigations designed to enhance cognitive function.

## Supplementary Information

Supplementary Information.
