# Author Correction: BDNF Val66Met gene polymorphism modulates brain activity following rTMS-induced memory impairment

**DOI:** 10.1038/s41598-022-05552-w

**Published:** 2022-01-17

**Authors:** Kilian Abellaneda‑Pérez, Pablo Martin‑Trias, Catherine Cassé‑Perrot, Lídia Vaqué‑Alcázar, Laura Lanteaume, Elisabeth Solana, Claudio Babiloni, Roberta Lizio, Carme Junqué, Núria Bargalló, Paolo Maria Rossini, Joëlle Micallef, Romain Truillet, Estelle Charles, Elisabeth Jouve, Régis Bordet, Joan Santamaria, Simone Rossi, Alvaro Pascual‑Leone, Olivier Blin, Jill Richardson, Jorge Jovicich, David Bartrés‑Faz

**Affiliations:** 1 Medical Psychology Unit, Department of Medicine, Faculty of Medicine and Health Sciences, University of Barcelona, C/ Casanova, 143, 08036 Barcelona, Spain; 2 Institute of Biomedical Research August Pi i Sunyer (IDIBAPS), Barcelona, Spain; 3 CIC CPCET Service de Pharmacologie Clinique et Pharmacovigilance, CHU Timone, AP-HM, Marseille, France; 4 Department of Physiology and Pharmacology “V. Erspamer”, Sapienza University of Rome, Rome, Italy; 5 Department of Neuroscience and Neurorehabilitation, IRCCS S. Raffaele, Rome, Italy; 6 Istituto di Ricovero e Cura a Carattere Scientifico (IRCCS) SDN, Naples, Italy; 7 Neuroradiology Section, Radiology Department, Diagnostic Image Center, Hospital Clinic of Barcelona, University of Barcelona, Barcelona, Spain; 8 Magnetic Resonance Image Core Facility (IDIBAPS), Barcelona, Spain; 9 INSERM, Inst Neurosci Syst, Aix Marseille Université, 13005 Marseille, France; 10 Inserm, CHU Lille, U1171, Degenerative and Vascular Cognitive Disorders, University of Lille, Lille, France; 11 Sleep Unit, Neurology Department, Hospital Clinic of Barcelona, Barcelona, Spain; 12 Dipartimento di Scienze Mediche, Chirurgiche e Neuroscienze, Brain Investigation & Neuromodulation Laboratory (Si‑BIN Lab), University of Siena, Siena, Italy; 13 Hinda and Arthur Marcus Institute for Aging Research and Deanna and Sidney Wolk Center for Memory Health, Hebrew SeniorLife, Boston, MA USA; 14 Department of Neurology, Harvard Medical School, Boston, MA USA; 15 Guttmann Brain Health Institute, Guttmann University Institute of Neurorehabilitation, Autonomous University of Barcelona, Badalona, Spain; 16 Neurosciences Therapeutic Area, GlaxoSmithKline R&D, Stevenage, UK; 17 Center for Mind/Brain Sciences (CIMEC), University of Trento, Trento, Italy

Correction to: *Scientific Reports* https://doi.org/10.1038/s41598-021-04175-x, published online 07 January 2022

In the original version of this Article Paolo Maria Rossini was incorrectly affiliated with ‘Hospital San Raffaele Cassino, Cassino, FR, Italy’. The correct affiliation is listed below.

Department of Neuroscience and Neurorehabilitation, IRCCS S. Raffaele, Rome, Italy.

The original Article has been corrected.

