# Supplementary material for: BDNF Val66Met gene polymorphism modulates brain activity following rTMS-induced memory impairment
Source: Sci Rep. 2022 Jan 7;12:176. doi: 10.1038/s41598-021-04175-x (PMC8741781; doi:10.1038/s41598-021-04175-x)
Supplement: Supplementary file 1 — Supplementary Information. [file 41598_2021_4175_MOESM1_ESM.docx]

***Supplementary Material:***

**BDNF Val66Met gene polymorphism modulates brain activity following rTMS-induced memory impairment**

**Materials and Methods**

**TMS protocol**

*Anatomical location of the stimulation target*

According to the FSL Harvard-Oxford cortical structural atlas, the location of this area corresponds to the middle frontal gyrus (24%), the precentral gyrus (20%), and the pars opercularis of the inferior frontal gyrus (19%).

**MRI protocol**

All participants underwent fMRI interleaved acquisitions [T2*weighted EPI scans, repetition time (TR) = 2,000 ms, echo time (TE) = 28 ms, 480 volumes, 34 slices, slice thickness = 3.5 mm, interslice gap = 15%, field of view (FOV) = 238 mm] during the performance of the memory recognition task. In addition, a high-resolution T1-weighted structural image was obtained for each subject with a magnetization prepared rapid acquisition gradient-echo (MPRAGE) three-dimensional protocol (TR = 2300 ms, TE = 2.98 ms, inversion time = 900 ms, FOV = 256 mm, 1-mm isotropic voxel). The T1-weighted structural acquisitions were used for TMS neuronavigation targeting, as described in the main manuscript. For all participants, MRI images were examined by a senior neuroradiologist for any clinically significant pathology (none found).

**Memory task**

*Memory task: Encoding phase*

Presentation® software package (Version 10.1, Neurobehavioral Systems, Inc., Berkeley, CA, www.neurobs.com) was used for implementing both encoding and recognition memory tasks. The encoding memory phase included a block design task lasting for 10 min and 25 s, and consisting of interleaving 3 blocks for LFC stimulation condition and 3 blocks for vertex stimulation condition. Condition order was counterbalanced across subjects. In each block, 12 emotionally neutral pictures were presented sequentially (50% indoor, 50% outdoor), resulting in 72 picture stimuli. Each picture trial was composed by a red cross (warning, 1 s), a picture (1 s), a green cross (answer warning, 1 s) and a jittered interstimulus interval (ISI; 4.06 ± 0.57 s, maximum ISI = 5 s, minimum ISI = 3 s). Subjects were asked to press “z” or “m” keyword keys to denote correspondence of indoor or outdoor pictures respectively at green cross appearance. To ensure similar subject’s familiarity with the rTMS protocol and TMS evoked skin sensitivity effects in all subjects, one short block (two trials) for each condition with no picture appearance was performed before to the main experimental task.

*Memory task: Recognition phase*

The recognition memory task was performed within the MRI scanner. This phase was undertaken ≈ 30 min after the TMS-encoding phase and lasted 16 min and 14 s. It consisted of the presentation of 48 new and 48 old pictures, from which were 24 previously encoded under LFC and 24 under vertex stimulation condition. Each picture trial was composed by a red cross (warning, 1 s), a picture (2 s) and a grey cross within time-jittering from 1 to 29 s (ISI; 6.67 ± 5.88 s). Following each picture presentation, subjects were asked to answer if they perceived it as previously seen or not (yes/no; i.e., old/new) using an MRI-compatible two-button keyboard (left for yes; right for no). In order to maximize statistical significance of the fMRI signal of recognition task and diminish subject habituation and expectation effects, Optseq2 software package (NMR Center, Massachusetts General Hospital, Boston, MA, USA) was used to deliver an optimized stochastic design that define the order and the exact timing for delivering each picture along the task with comparable fMRI signal regarding conditions (LFC, vertex, and new pictures).

**Genotyping**

Saliva samples were collected utilizing the BUCCALAMP™ DNA EXTRACTION KIT. The region of the BDNF Val66Met polymorphism was amplified using a real-time polymerase chained reaction (RT-PCR) cycler 7900HT (Life Technologies) and a TaqMan 5′ nuclease assay kit (Life Technologies). RT-PCR was made using 0.5 uL of 10X PCR MasterMix, 0.25 uL 40X TaqMan assay in a final volume of 5 uL. RT-PCR runs included a denaturing step of 10 min − 95 °C followed by 40 PCR cycles (1 min 95 °C, 1 min 60°C). Val/Met and Met/Met subjects were considered as the ‘Met group’ (i.e., homozygotes and heterozygotes Met carriers). For the Val/Val individuals (i.e., Val homozygotes), ‘Val group’ was used as their label group. The distribution of the genotypes in our sample was in Hardy-Weinberg equilibrium (*p* = 0.26, Chi-squared test). Specific written informed consent was provided for acquisition and assessment of genetic data.

**fMRI data**

*fMRI data preprocessing*

fMRI data preprocessing included non-brain tissue removal, motion correction, spatial smoothing with a Gaussian kernel of 6 mm of Full Width at Half Maximum (FWHM), temporal filtering with a high pass filter of 90 s and a linear registration to a standard template. Moreover, the head motion parameters estimated by MCFLIRT^1^ were included as confounding explanatory variables in our model.

*fMRI first-level processing*

First-level analysis^2^ were customized taking into account the individualized performance. Data were fit to a FILM general linear model (GLM) with local autocorrelation correction^2^. Two events categories were used: hits at frontal cortex condition (‘hits frontal’; HF) and hits at cranial vertex condition (‘hits vertex’; HV). Each individualized FILM GLM contained the task events with a doble gamma convolution of the hemodynamic response function. In this GLM, two regressors (i.e., HF and HV) and their temporal derivatives and temporal filtering were modeled. Subsequently, we defined three contrasts of interest combining the previous two regressors: (1) mean HF; (2) mean HV; and (3) HF>HV.

**Statistical analysis**

*Sanity check analyses*

The variables of center, age and educational attainment were compared between genetic groups regardless the experimental condition using independent-samples t-tests and chi-squared tests as control sanity checks.

*Responder vs. non-responder analyses*

In the present investigation, we further classified our participants as TMS responders or non-responders. Subjects were classified as “responders” if their behavioral responses to rTMS were equal or lower than 0 (i.e., had any negative value) when subtracting the vertex to the LFC stimulation (i.e., they had lower scoring during LFC as compared to vertex). Otherwise, they were classified as “non-responders” (i.e., they had equal or higher scoring during LFC as compared to vertex). Differences in subjects frequency distribution considering BDNF Val66Met gene polymorphism (i.e., Val vs. Met) and TMS responsiveness (i.e., “responders” vs. “non-responders”) both at encoding and retrieval were calculated with chi-squared tests.

**Results**

**Cognitive performance**

*Sanity check results*

As a sanity check, we found that age, center, and educational attainment did not differ between BDNF Val66Met gene polymorphisms (age: *t_(41)_ = -*0.186, *p =* 0.853; center: *χ^2^* = 2.484, *p* = 0.115; educational attainment: *χ^2^* = 1.293, *p* = 0.255).

|  | | **N** | **Mean** | **SD** | **Min** | **Max** |
| --- | --- | --- | --- | --- | --- | --- |
| **Accuracy: LFC** | Val group | 22 | 94.57 | 5.32 | 83.33 | 100.00 |
|  | Met group | 18 | 97.07 | 3.23 | 88.89 | 100.00 |
|  | All subjects | 40 | 95.69 | 4.62 | 83.33 | 100.00 |
| **Accuracy: Vertex** | Val group | 22 | 97.73 | 2.37 | 91.67 | 100.00 |
|  | Met group | 18 | 95.83 | 3.96 | 88.89 | 100.00 |
|  | All subjects | 40 | 96.87 | 3.28 | 88.89 | 100.00 |
| **Accuracy’s RT: LFC** | Val group | 22 | 827.73 | 215.48 | 478.56 | 1213.73 |
|  | Met group | 18 | 771.31 | 229.95 | 443.79 | 1432.47 |
|  | All subjects | 40 | 802.34 | 221.04 | 443.79 | 1432.47 |
| **Accuracy’s RT: Vertex** | Val group | 22 | 822.67 | 205.05 | 417.17 | 1223.92 |
|  | Met group | 18 | 714.84 | 186.75 | 411.79 | 1112.43 |
|  | All subjects | 40 | 774.15 | 201.97 | 411.79 | 1223.92 |
| **Hits: LFC** | Val group | 24 | 72.22 | 17.92 | 29.17 | 100.00 |
|  | Met group | 19 | 73.90 | 15.14 | 50.00 | 100.00 |
|  | All subjects | 43 | 72.97 | 16.58 | 29.17 | 100.00 |
| **Hits: Vertex** | Val group | 24 | 78.99 | 12.97 | 50.00 | 95.83 |
|  | Met group | 19 | 78.07 | 13.74 | 50.00 | 100.00 |
|  | All subjects | 43 | 78.59 | 13.16 | 50.00 | 100.00 |
| **Hits’ RT: LFC** | Val group | 24 | 1207.88 | 337.09 | 794.69 | 2563.27 |
|  | Met group | 19 | 1091.63 | 166.10 | 840.92 | 1376.61 |
|  | All subjects | 43 | 1156.52 | 278.32 | 794.69 | 2563.27 |
| **Hits’ RT: Vertex** | Val group | 24 | 1200.69 | 350.80 | 784.76 | 2468.14 |
|  | Met group | 19 | 1070.18 | 144.43 | 870.77 | 1311.57 |
|  | All subjects | 43 | 1143.02 | 283.95 | 784.76 | 2468.14 |

**Table S1.** Behavioral data is displayed for encoding and retrieval and considering the two experimental conditions (LFC and vertex) and the two BDNF Val66Met groups (Val and Met). For encoding data, accuracy refers to the percentage of pictures correctly categorized as indoor or outdoor, while accuracy’s RT refers to the time elapsed from the presentation of a picture to the subsequent response (in milliseconds). For retrieval data, hits refer to the percentage of correctly recognized pictures, while hits’ RT refers to the time elapsed between picture appearance and yes/no motor response (in milliseconds). Abbreviations: LFC, left frontal cortex; RT, reaction time. Please, note that 40 subjects were available for encoding and 43 for retrieval analyses.

*Responder vs. non-responder analyses*

During the encoding, in the Val group, out of the 22 subjects, 13 individuals were “responders”, while 9 were “non-responders”. In the Met group, out of the 18 participants, 5 individuals were “responders”, while 13 were “non-responders”. These differences were statistically significant (*χ^2^* = 3.922, *p* = 0.048). During the retrieval, in the Val group, out of the 24 subjects, 14 individuals were “responders”, while 10 were “non-responders”. In the Met group, out of the 19 participants, 10 individuals were “responders”, while 9 were “non-responders”. These differences were not significant (*χ^2^* = 0.140, *p* = 0.708). In the subsequent figure (Fig. S1), it can be observed group TMS effects on cognition including individual values (left) as well the individual relationships between both experimental conditions (right) in each genetic group.

**Fig. S1.** Individual TMS effects on cognition as a function of BDNF Val66Met gene polymorphism. Cognitive performance results considering accuracy during encoding in A) Val group and B) Met group as well as hits during recognition in C) Val group and D) Met group. Abbreviations: LFC, left frontal cortex.

**fMRI findings**

*Genetic differences during HF*

Additionally, we further explored if in the mean of activity maps during HF and HV, there were differences between the genetic subgroups. As these results were influenced by center, these sub-analyses are specifically presented controlling for this factor. We detected group differences (Val vs. Met) for the mean HF contrast of interest, as Val group exhibited more activation than Met group in distinct imaging clusters. The first cluster comprised the pre- and post-central gyri. The second cluster encompassed the inferior division of the lateral occipital cortex, the occipital fusiform gyrus and the occipital pole. Finally, a third imaging cluster covered the anterior and posterior divisions of the supramarginal gyrus, the posterior division of the superior temporal gyrus, and the planum temporale (Fig. S1A). No significant group differences were detected for the mean HV contrast. Furthermore, additional non-imaging analyses revealed that superior individual mean BOLD values within the significant result derived from the mean HF & Val>Met contrast (Fig. S1A), were correlated with greater performance for hits (*r* = 0.524, *p* = 0.009, Fig. S1B). These results survived when additionally controlling for center (*r* = 0.525, *p* = 0.010). No significant correlations were detected here for the Met subgroup (without controlling for center: *r* = 0.253, *p* = 0.297; controlling for center: *r* = 0.203, *p* = 0.420).


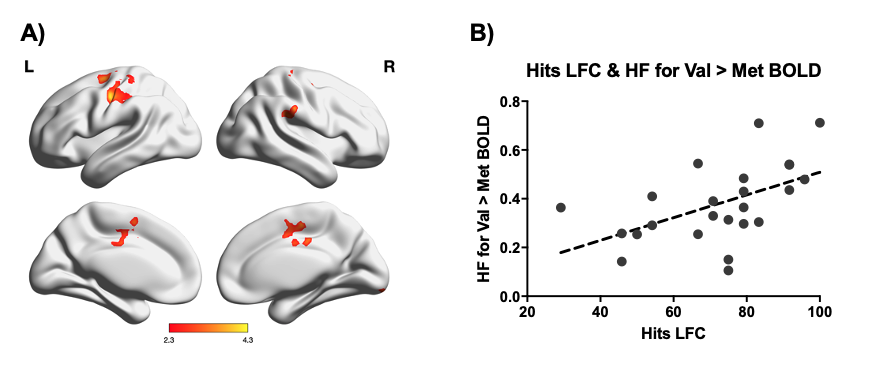


**Fig. S2.** A) ﻿Brain activity maps regarding mean HF for Val>Met imaging contrast. B) Scatter plot showing the Pearson correlation between hits performance during LFC condition and mean ﻿BOLD signal values within the ROIs displayed in A, only considering the Val group. Abbreviations: LFC, left frontal cortex; HF, hits frontal cortex; HV, hits cranial vertex; ﻿BOLD, blood oxygen level dependent.

*Stimulation area comparison*


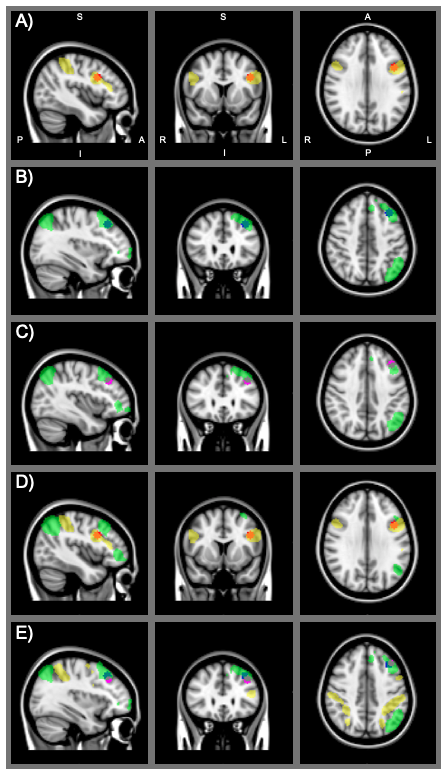


**Fig. S3.** Topographical allocation of the targeted TMS region in the current study as compared to similar stimulation areas within standard brain networks from Shirer et al^3^. A) LFC targeted area (in red; MNI coordinates: X = - 42; Y = 10; Z = 30, according to Martin-Trias et al.^4^) fall within the dorsal attention network (DAN; in yellow; mainly comprised by the intraparietal sulcus and the frontal eye fields). B) F3 location according to Keeser et al.^5^ (in blue; ﻿MNI coordinates: X = −34; Y = 26; Z = 44) fall within the left-executive control network (LECN; in green). C) F3 location according to Rojas et al.^6^ (in pink; MNI coordinates: X = −38; Y = 28; Z = 38) partially fall within the LECN. D) LFC targeted area (in red) displayed with the DAN (in yellow) and LECN (in green) to corroborate its DAN-specific overlapping. D) F3 according to Keeser et al.^5^ (in blue) and Rojas et al.^6^ (in pink) displayed with the DAN (in yellow) and LECN (in green) to corroborate its LECN-specific overlapping.

**References**

1. Jenkinson, M., Bannister, P., Brady, M. & Smith, S. Improved optimization for the robust and accurate linear registration and motion correction of brain images. *NeuroImage* **17**, 825–841 (2002).

2. Woolrich, M. W., Ripley, B. D., Brady, M. & Smith, S. M. Temporal autocorrelation in univariate linear modeling of FMRI data. *NeuroImage* **14**, 1370–1386 (2001).

3. Shirer, W. R., Ryali, S., Rykhlevskaia, E., Menon, V. & Greicius, M. D. Decoding subject-driven cognitive states with whole-brain connectivity patterns. *Cereb. Cortex* **22**, 158–165 (2012).

4. Martin-Trias, P. et al. A study of BOLD reproducibility: visual encoding, memory and resting state in Organization for Human Brain Mapping, Hamburg, Germany. (2014).

5. Keeser, D. *et al.* Prefrontal transcranial direct current stimulation changes connectivity of resting-state networks during fMRI *J. Neurosci.* **31**, 15284–15293 (2011).

6. Rojas, G. M. *et al.* Study of resting-state functional connectivity networks using EEG electrodes position as seed. *Front. Neurosci.* **12,** (2018).
